# Social norms and social opportunities: a qualitative study of influences on tobacco use among urban adolescent girls in Ghana

**DOI:** 10.1186/s12889-024-20413-z

**Published:** 2024-10-28

**Authors:** Lois N.A. Aryee, Sara V. Flanagan, Lydia Trupe, Morgan Yucel, Jana Smith

**Affiliations:** 1 ideas42, Accra, Ghana; 2 ideas42, New York, NY USA

**Keywords:** Adolescent girls, Tobacco, Smoking, Social norms, Ghana

## Abstract

**Background:**

Tobacco use is a global public health threat and a leading preventable cause of death in the world. While cigarette use among youth has been decreasing in high-income countries, low- and middle-income countries have contrastingly high rates of adolescent smoking. In Ghana, smoking prevalence is lower than in other parts of the African continent and more common among men than women; however the gender gap in tobacco use among adolescents has narrowed, with shisha use among girls recently surpassing boys. These trends toward increased relative use among adolescent girls are a cause for concern and highlight the need for interventions to prevent and/or reduce tobacco use in this population.

**Methods:**

We conducted in-depth interviews with girls aged 13–20 (*n* = 148) and their parents (*n* = 7) in Ghana’s largest two cities – Accra and Kumasi, to explore the physical, social, and psychological context underlying smoking perceptions and behavior.

**Results:**

We identified 11 key insights into influences on smoking behavior, organized into the broader themes of perceptions of smoking, environmental factors, and internal factors. Findings underscore that perceptions around smoking are very much driven by girls’ social world, which is generally small and parent-centric for non-smokers at younger ages but can start to expand as girls get farther along in school or leave school entirely. After what may have been a sheltered adolescence, many older girls in Ghana look for ways to express their independence in decision-making and a few may use their developing agency to rationalize limited or infrequent tobacco use if they perceive the benefits to them outweigh the potential consequences.

**Conclusions:**

Our findings highlight strong social influences on girls’ perceptions, exposure to opportunities, and decision-making in ways that can often be protective against smoking, particularly at younger ages, but may also leave girls unprepared to manage challenging situations later in life. Understanding this context in Ghana will be important for any future programming aiming to prevent smoking initiation among adolescents.

**Supplementary Information:**

The online version contains supplementary material available at 10.1186/s12889-024-20413-z.

## Background

Tobacco use is a global public health threat and a leading preventable cause of death in the world [1] It is a risk factor for several non-communicable diseases, including cancer, cardiovascular diseases, and respiratory diseases [2], and causes over 8 million deaths per year, including exposure to secondhand smoke [1]. Girls and women who smoke additionally face increased risk of reproductive health complications including cervical cancer, miscarriages, premature birth, and low birth weight infants [3]. In recent decades, the burden of tobacco use has shifted towards low and middle-income countries (LMICs), where 80% of the world’s tobacco users currently live [4]. Tobacco-attributable deaths are also projected to double in LMICs between 2002 and 2030 [5], including in sub-Saharan Africa. These trends are the result of various factors, including urbanization, westernization of lifestyles [6], income growth, and the aggressive expansion of and advertising from the tobacco industry [7]. Increased regulation and restrictions on the tobacco industry in developed countries have led them to pursue new markets in LMICs, where there is relatively less legislation against advertising and distribution [8]. A recent situational analysis in Ghana confirmed the while the country has made progress in establishing a regularly framework for tobacco control in line with global guidance, several priority areas need more action, including developing gender-specific and age-specific interventions to minimize future uptake [9].

Although the prevalence of smoking among men has been historically higher than for women, this gap has narrowed over time globally [10]. A similar pattern is emerging in Sub-Saharan Africa, where smoking prevalence among girls has recently become as high as for boys [5]. In several countries, especially those with low human development indices, adolescent girls are using tobacco at higher rates than adult women [11]. In Ghana, smoking prevalence is much lower than in other parts of the African continent [12], but as elsewhere can lead to declines in household well-being as tobacco and alcohol expenditures reduce spending on food, housing, and health needs [13]. Men are still significantly more likely to smoke than women [14], but tobacco use is increasing among young women. A 2012 study found that adult male use was 10% higher than adult female use, but the gap between school-going adolescent males and females (ages 11–17) was much narrower – 2.3% for ever-smokers and 1% for current smokers [15]. Non-cigarette tobacco products are contributing to this narrowing gender gap [16]; shisha use is now more prevalent among girls than boys in Ghana [17]. These trends suggesting increasing tobacco use relative to boys are a cause for concern and highlight the need for interventions to prevent and/or reduce tobacco use among young female adolescents.

Adolescence is a critical transition period, and tobacco exposure at such a young age increases risks for lifelong addiction. Studies show that a majority of deaths among adults are a result of behaviors that started in adolescence [18], and whereas most adults who smoke developed the habit during their teenage years [19], people who avoid smoking in adolescence are unlikely to ever start use [20]. While use of cigarettes among youth has been decreasing in high-income countries [11], LMICs have contrastingly high levels of adolescent smoking [21]. However, much of the research into the underlying drivers and determinants of tobacco use among adolescents comes from high-income countries. Global studies show that adolescents are prone to engaging in risky behaviors [22] and are susceptible to peer pressure and other social influences [23], making them vulnerable to trying and using tobacco products [24]. They also find smoking by adolescents to be associated with age [20]; exposure to family, friend, and peer tobacco use [25]; alcohol and illegal drug use [8]; and general exposure to secondhand smoking [7]. Tobacco use is higher for adolescents from lower socioeconomic backgrounds and lower for adolescents from two-parent households [26]; however, adolescents with more access to disposable income are also more likely to smoke [8, 27]. In high-income countries, tobacco use among teenage girls has also been linked to low self-esteem [28, 29], with adolescent girls reporting use for weight management [30] and for expressing independence [31], as well as for relaxation [32] and for coping with stress [33].

In Ghana, several descriptive studies on the prevalence and susceptibility of smoking among adolescents find similar patterns as have been seen globally ─ including correlations with age [15], parental smoking [34], gender [35], pocket money and socioeconomic status [36], exposure to tobacco advertisement [34], alcohol and marijuana use [35], and exposure to friends who smoke [37]. The 2017 Ghana Youth Tobacco Survey found that 2.8% of Ghanaian 13-15-year-olds (3.2% boys and 2.3% girls) currently smoke cigarettes, and 1.3% (0.4% boys and 1.7% girls) smoke shisha [38]. Significant regional differences in youth tobacco use reflect overall tobacco use, with higher prevalence observed in the Savannah and Northern regions which tend to have lower socioeconomic status (SES), compared to the Central and Southern parts of Ghana [36].

Very few studies, however, have qualitatively explored the mechanisms that underlie adolescent smoking decisions and behaviors in Ghana, especially among female youth. A more nuanced understanding of the behavioral influences will be important for designing early intervention strategies that are responsive to this population and context. For example, conversations among peers have been found to mediate the effects of mass media campaign exposure in Ghana [39]; better understanding the social dynamics underlying perceptions of smoking could help both to improve the effectiveness of future communication interventions and consider other pathways for impact. Our study addresses this gap in the literature, using a behavioral science lens to investigate the social and physical context of adolescent girls and how these environmental features may influence decisions and actions around tobacco use.

Here we present formative research conducted prior to the impact evaluation of a relaunched multi-media social marketing campaign aimed at preventing tobacco use among adolescent girls [40]. Our goal was to understand the context of tobacco use among teenage girls and to explore the behavioral mechanisms and pathways by which girls are exposed to and/or introduced to tobacco in Ghana. The formative research enabled us to understand how girls form perceptions and attitudes toward smoking, as well as the factors that influence their smoking (or non-smoking) behavior. The findings were used to refine the theory of change for the social marketing campaign and informed the creation of survey instruments for the impact evaluation.

## Methods

### Study setting

We conducted in-depth interviews in Ghana’s two largest cities – Accra and Kumasi, where the campaign was planned to be implemented and evaluated, in the Greater Accra and Ashanti regions respectively. Both cities are economically vibrant, with Accra as the capital city and Kumasi known for hosting one of the largest markets in West Africa. Accra is more cosmopolitan and culturally diverse—a melting pot of people from various parts of the country, whereas Kumasi is more homogeneous with more adherence to traditional practices, although balanced with modern lifestyles. Gender norms in Kumasi also tend to be more traditional than in Accra.

Field work was conducted over three rounds: December 2020, February 2021, and February 2022 to allow for iteration and additional investigation based on emerging themes and to provide context for the evaluation. The results reported here reflect findings across all three rounds. Some of the interviews were conducted in schools; others were conducted in the homes of teenage girls or in their communities. A few qualitative interviews with girls and all parent interviews were conducted over the phone (either the participants’ or a family member’s) during the second round of data collection.

### Study context

This exploratory study was conducted to inform the evaluation of a multimedia youth anti-smoking and girls’ empowerment campaign and was guided by the theory of change developed by the program’s designers and implementers. The campaign is intended to create an environment of social identity and inclusion that encourages girls to stay true to their own health and well-being, strips away the aspirational aspects of smoking, and increases ability to resist social pressure [40]. Investigators initially adjusted the program’s theory of change [40] based on their experience applying the behavioral design approach, which leverages insights from behavioral economics, social psychology, and human-centered design, among other disciplines, to design and test solutions that reshape environments in support of positive behavior change [41]. A critical step of this approach is to diagnose the behavioral reasons and context contributing to a problem through examining the range of features within one’s environment—physical, social, psychological—that may influence behavior. This entails exploring the applicability of a wide range of behavioral principles drawn from existing behavioral theory and models along with reviewing past studies to identify the key behavioral channels and barriers.

The resulting behaviorally-informed theory of change [42] laid out the features of the intervention to be implemented and the causal pathways by which those mechanisms may affect outcomes of interest. The theory of change covered pathways and opportunities by which teenage girls could be exposed or introduced to tobacco use, and the features of their environment (relationships, beliefs, societal factors, etc.) that could lead them to take up or refuse to try tobacco products. This refined theory of change guided the development of interview guides and the coding framework.

### Study participants

Data collection included 148 qualitative interviews with adolescent girls aged 13 to 20 (and an additional 27-year-old sibling with whom we spoke in an effort to speak to more smokers). We also interviewed 7 parents of adolescent girls to better understand the mechanisms by which parents influence their smoking behavior.

A purposive sample of teenage girls was selected to participate in the study, recruited from the Madina and Kisseman neighborhoods of Accra and the Tech and Oduom neighborhoods of Kumasi. The sample was selected to represent a diversity of perspectives and experiences by targeting a mix of in-school vs. out-of-school girls, public vs. private school attendees, younger (aged 13-16) vs. older (aged 17-19) teenage girls, and girls from both lower and higher SES backgrounds. The average age of girls interviewed was 15. Most were in school, although about one in five had either already graduated or dropped out. Most reported living with one or both parents; the rest lived with grandparents or other relatives. Nine girls reported personal experience with smoking, either of cigarettes or shisha. Parents of girls aged 13–19 were selected to represent a mix of lower and higher SES.

### Processes

Qualitative interviews explored social influences, knowledge about and experience with tobacco, resources and competencies for decision-making, and extracurricular activities [see Additional file 1]. The guides were refined during the second round of data collection with additional probes to explore more extensively the influence of parents and boys in girls’ smoking behavior and decision-making, after they emerged as strong themes during the first round. The guides for the third round of data collection were more narrowly focused on understanding girls’ personal experiences with smoking, their understanding of and exposure to tobacco products, as well as the influences of COVID-19 restrictions on social activity, as additional context for program implementation and to complement the initial findings. The program theory of change was then revised a final time to reflect the evidence generated from this study.

Girls were identified and scheduled for interviews by our partner local research firm, based on rough targets set by the investigators. Some girls were identified and recruited through door-to-door visits. Others were approached in the community and were informed by the research team about the study. In a few cases, we targeted smokers specifically and conducted interviews with them in clubs, pubs, slums, or in other locations where girls were likely to be found smoking. Girls who were identified in such locations were approached and asked whether they smoked, and if they reported doing so, were recruited by the research team. Some girls were interviewed on the same day as they were recruited. Others were consented and interviewed by the study investigators between 1 and 5 days after they were identified and recruited by partners. When parents and girls were recruited for interviews to be conducted by phone, informed written consent was first obtained in person by the partner research team.

All qualitative interviews during the first two rounds were conducted in English by three female members of the study team, one Ghanaian and two American, with interpretation to the local language, where needed, provided directly by the Ghanaian investigator. Working in pairs, the researchers transcribed anonymized responses live during each interview into a password-protected spreadsheet. Third-round interviews were conducted by Ghanaian female enumerators from the local research firm and transcribed based on handwritten notes and audio recordings.

Participants were interviewed only once for about 60 min, in locations where privacy could be maintained, outside of the earshot of others. The researchers did not have any relationship with participants prior to conducting the interviews. Parents of unemancipated girls under 18 years of age were first taken through a parental informed consent process and provided written consent before girls were approached for their assent. All respondents were also taken through a voluntary informed consent process to explain the details of the study and they provided written consent or assent before interviews were administered. Our research was approved by an international Institutional Review Board (IRB), Innovations for Poverty Action. We also obtained permission from a local IRB body – the Ghana Health Service Ethics, Research, and Management Department.

### Analysis

We conducted an exploratory thematic analysis [43] of interview responses, updating and revising initial themes about social, environmental, and psychological influences on tobacco use as new themes emerged across rounds to capture all relevant insights. We developed an initial coding framework based on themes in the interview guide that were drawn from the program’s theory of change. Members of the research team used the framework to individually code each transcript, with several transcripts double-coded to ensure consistency among the research team. Once all transcripts were coded, data relevant to hypotheses and sub-themes within each broad theme was excerpted into Microsoft Excel and Airtable and synthesized to draw key insights based on the strength of the evidence and with consideration of the behavioral literature. This process highlighted the most relevant factors that adolescent girls reported were influential to tobacco decisions and actions. Summary findings and interpretation were shared with local partners for validation and contextual grounding.

This manuscript follows the O’Brien et al. Standards for Reporting Qualitative Research [44].

## Results

**Fig. 1:**
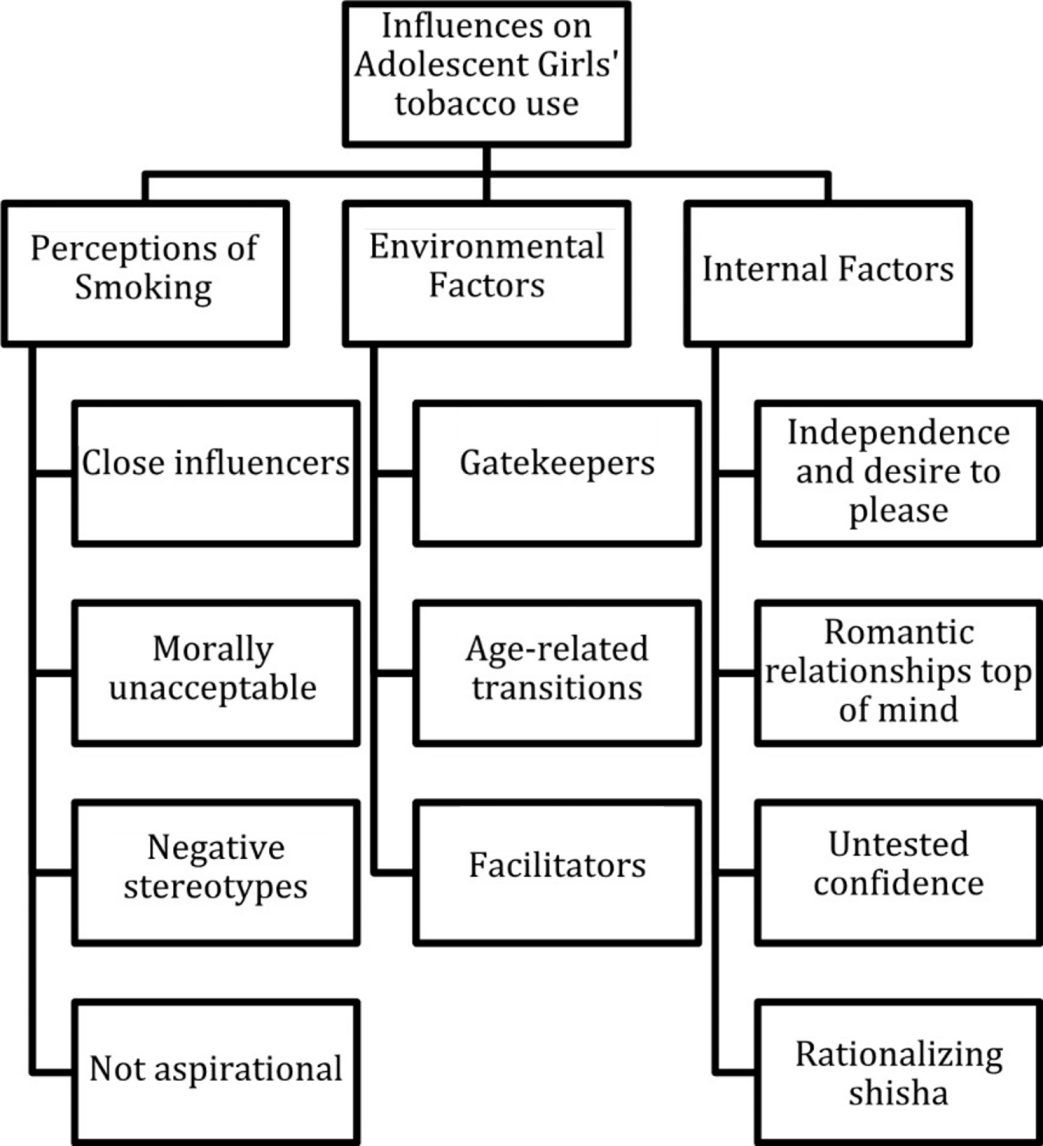
Thematic tree of qualitative insights

We identified 11 key insights through our formative research (Fig. 1), organized below into 3 categories: perceptions of smoking, environmental factors, and internal factors, which align with the broad themes drawn from the program theory of change and are named in accordance with other literature on the influences on youth tobacco use. Here we define “Perceptions of smoking” to include the social influences and perceived norms contributing to girls’ beliefs and attitudes about smoking and smokers. “Environmental factors” include the physical and social environments and factors that shape girls’ access to tobacco and opportunities for smoking. “Internal factors” include psychological factors that may influence decision making, including how girls perceive and respond to the smoking opportunities presented to them. In this study, cigarettes and shisha were the primary forms of tobacco participants mentioned as in use by adolescents.

### Perceptions of smoking

1. Close influencers: Girls’ perceptions of smoking are influenced more by their social networks of parents, close friends, and romantic partners, rather than more remote influences like celebrities or “cool” kids.

Adolescent girls reported spending most of their time with their closest friends when they are at school and with parents and family when they are out of school. They therefore rely on these people for guidance on how to behave – both through explicit advice and by observing how they live. Girls also listen most to people they trust, and these tend to be people with whom they are in the closest relationships. They seek information and advice about social issues from these people. Additionally, most teenage girls feel an obligation to listen to their parents’ advice because of the sacrifices they have seen their parents make for them. They also trust that their parents want what is best for them and that their parents have more experience which they can learn from. Girls may admire celebrities like musicians and actors but only want to emulate them when the celebrities’ values/actions match theirs.

*[I listen to] my parents’ [advice most*,* because] they gave birth to me*,* take good care of me*,* and they came to this world before me*,* so they know the right things and the bad things.* (14-year-old non-smoker)

*I go to my parents for advice because they have gone ahead of me*,* they have experienced things that I have not experienced…they are trying to help me avoid the mistakes they have made. I like to listen to people like that; those who have had experience.* (20-year-old non-smoker)

*My friends influence me. I did not use to do all these things before*,* like smoking. When I joined this group of friends*,* they influenced me… I listen to people who care about me and love me because I know they want the best for me. And also people I respect.* (19-year-old smoker)

Girls involved in romantic relationships can face pressure from their partner to match their own preferences and behavior – whether in favor of smoking or not.

*[Girls my age most often buy cigarettes] when they start doing boyfriend-girlfriend because when your boyfriend is bad or likes smoking you will just say let me just join him after all he’s my boyfriend and I must follow his steps.* (14-year-old non-smoker)

*The boy I was dating was the one who told me to stop these things*,* like partying*,* so I listened to him.* (19-year-old former smoker)

2. Morally unacceptable: Most girls have never tried smoking and, consistent with their parents and other non-smoking friends, perceive it as morally wrong and associated with other negative behaviors and health risks. Only girls who smoke are supportive of friends doing the same.

Girls are aware that smoking is negatively perceived due to the health risks like heart problems and cancer that are warned of in advertisements and on cigarette packaging and based on comments they have heard other people make. Most girls believe that smoking affects the body, mind, and behavior of users negatively. Smoking is also seen by girls and their parents as morally unacceptable, both from religious and from social norms perspectives. Smoking is linked to vices like theft, drug use, and in a few cases, prostitution, and many girls reported that people who smoke are not respected within the community.

*When they [smokers] get money or even if they don’t have money*,* they borrow money*,* or when they don’t have the money*,* they go and steal.* (14-year-old non-smoker)

*When people use shisha*,* adults see them as very bad children and they see them as people who will not have a very good future. Even young kids cannot respect you if you smoke or if your parents smoke.* (19-year-old former smoker)

Religion is culturally prominent in Ghana and when describing people who did not smoke, girls sometimes described them as “God fearing.” Church activities like singing in choir were one of the few social activities that parents often allowed their daughters to participate in, given their perceived positive influence. One girl specifically mentioned Islam as the reason why she did not smoke. Only the minority of girls who smoke tend to view others who do the same favorably.

3. Negative stereotypes: Girls perceive smokers as wayward individuals without involved or responsible parents. In their minds, this includes both people who hang around the streets in slum areas, drunk or idle, or privileged teenagers with disposable money and no oversight.

Both girls and parents remarked that Ghanaian parents tend to heavily control the activities that their children engage in. As a result, they share the perception that people from responsible homes will not have access to smoking opportunities. Smokers are therefore viewed as having parents who are not responsible, present, or involved.

*If a young boy is seen smoking*,* people will think his parents did not advise him. Because if your parents advised you*,* you wouldn’t do that. A girl who smokes is seen as not having parents or not living with her parents or not brought up well.* (16-year-old non-smoker)

Smoking is also associated with irresponsibility on the part of the smoker, due to the negative ways in which it affects the user’s health and social outcomes. People who smoke are often not respected in society, and are shunned by their friends and families, so a person who smokes voluntarily is viewed as very rebellious and in some cases, even as an outcast.

Sometimes, smoking is perceived as a coping mechanism for people who are in unpleasant situations or are going through negative circumstances. For example, when girls are asked why other people smoke, they mention things like the fact that smokers may be from low SES backgrounds, going through heartbreak, or experiencing other forms of anxiety.

*People use cigarettes because they have heard from others that it makes you happy*,* and they also want to be happy. And sometimes also*,* smoking cigarettes gives them energy to do the work that they have to do.* (20-year-old non-smoker)

Smokers can also be from privileged backgrounds, if they have access to pocket money and little parental guidance. This is especially true for girls who smoke shisha, which is viewed as more expensive than cigarettes. People from privileged backgrounds may be viewed as having more freedom to take certain risks, because they are not as worried about the consequences – they and their parents have the means to make a life for themselves, even if they get involved in activities that could lead to negative social and health outcomes.

*Rich people [use shisha]*,* because they have the money to buy shisha. Because if they join a group*,* they also want to show that they are there [cool]; [that] they know what’s up.* (13-year-old non-smoker)

4. Not aspirational: While smoking may be considered “cool,” in a rebellious way, it is not seen as aspirational for most non-smoking girls given such negative perceptions of smoking among their more influential close friends and parents.

Many girls mentioned that smokers are likely to be popular or seen as “cool.” These perceptions were mainly in the context of more privileged smokers who were in school – i.e., in reference to their peers who were likely to have tried or used tobacco, or shared by smokers themselves.

*Shisha is good to smoke because it taste like toffee and usually it’s the slay queens and rich people who can afford because it’s very expensive. People also admire you when they see you smoking shisha in that small cute bottle in your hand*,* it’s like you are a slay queen or rich and the guys feel you because you are current.* (18-year-old smoker)

Given the visibility of these peers, about a third of interviewed girls perceived that smoking was “common” among girls their age, contrary to the actual low smoking rates in this group, and despite having no close friends who smoke. Yet, even though girls described these visible smokers as “cool,” they just as often also described them as “bad.” Non-smoking girls did not express any desire to emulate such people, given that they do not consider them as part of their social circles or having shared values.

*A lot of them [smokers] want to attract attention. So*,* they always do things that everybody will see and things that are bad.* (16-year-old non-smoker)

*Some do it for fun because it’s seen as cool – you know what’s up; you’re ‘gang’ [cool]; it’s like a fantasy. People who do it feel [like] they‘re ‘gang’*,* like “I know what’s up.”* (17-year-old non-smoker).

### Environmental factors

5. Parents as protectors and gatekeepers: Teenage girls of all ages rely heavily on their parents for advice and guidance, whereas parents tend to actively restrict the social life of their children to protect them from negative influences.

Girls consistently reported that many parents do not allow their daughters to go out, except for school, church or to run errands due to fear for their safety and concerns about exposure to negative influences from others. Also, many parents expect their children to stay at home and help with household chores when they are not in school. These restrictions were confirmed or implied by almost all of the parents that were interviewed. Parents may relax their restrictions gradually as girls get older, and may be slightly more flexible with their sons, but respondents agreed that most parents generally exert a lot of control over the movement of their daughters regardless of age.

*At home – [she is] washing*,* cooking*,* cleaning. Outside the home [she is] going to the market to buy food stuff for the house and sending the little one to school. From my place to the market is not all that far so I allow her to go but beyond that I don’t allow her to go.* (Mother of 14-year-old)

*My friends and I don’t go out*,* no not really. I’ve never actually been to a pool party or club. Because most of the time*,* my parents won’t allow me… they think that the time is wrong*,* most of the time it’s in the evening so… so it’s for your safety.* (18-year-old non-smoker)

Many parents do not encourage their daughters to make or spend time with friends due to the fear of negative influences, including distraction from important chores and studies.

*Making too many friends - I don’t like it. When you have too many friends*,* you get distracted*,* you don’t do what is expected of you.* (Mother of 14-year-old)

6. Age-related transitions: Exposure to smoking, and in turn likelihood of experimenting, increase with age as girls gain more freedom from parents to attend social events, attend boarding school and gain exposure to girls from more diverse backgrounds, and have more exposure to digital media.

Some girls described being given more freedom as they get older. Parents may gradually relax their restrictions on movement, and older girls may be more involved in activities outside the home, which enables them to spend less time with their parents and more time with friends. Many girls in urban Ghana, usually between ages 15–16, enroll in a boarding school for their Senior High School (SHS) Education. Interview participants described suddenly spending a lot of time with friends and having very limited interactions with their parents. Girls may also attend different boarding schools than their neighborhood friends, giving them good reason to organize meetups during the holidays. Even when they attend day schools, many girls are exposed to activities that they may not have been exposed to in Junior High School (JHS), like organized entertainment activities, parties, dances, etc. Girls typically attend high schools outside of the communities or cities they reside in and meet girls from different regions and diverse upbringings who may introduce them to different lifestyle choices and peer networks.

*In JHS*,* we were young*,* so we just talked about things. But in SHS*,* we went out a bit to the KFC or seaside or club to chill a bit.* (18-year-old non-smoker)

*Yes I did hang out with all my friends at the beach*,* club and pubs and met new friends as well when I went out. Most of them are guys who are older than myself and my friends.* (18-year-old smoker)

Additionally, girls described beginning to hide more from their parents as they grow older (from JHS to SHS), particularly about their relationships with boys. Furthermore, when girls are in JHS, parents typically only give their daughters lunch money, but in SHS, parents will provide more pocket money and trust their daughters with more responsibilities like paying for certain expenses including their school fees, lodging, and upkeep. In addition to paying for necessities, girls typically also use the money to pay for cellular data and clothes. Parents of SHS girls sometimes give “generous” disbursements to their daughters to ensure they are not tempted to seek boyfriends for money.

Older adolescent girls were more likely to have phones than younger adolescent girls, and this may give them access to ideas, information, images, and trends that they may not have been exposed to when they were younger, including those suggesting that tobacco use may be more common among peers. Girls who leave home or school and experience more freedom from an early age are also more likely to be confronted with smoking earlier as their social circles change.

*I began using it [shisha] long ago. I was very young*,* maybe about 13. It was when I stopped going to school. I joined a group of friends who were doing it*,* so I also started doing it… they told me to try it*,* and that’s how I started*. (19-year-old smoker)

*It was through friends that I was introduced [to cigarettes]. Because I was not in school*,* I went to sit next to them. They took me to a club and we bought Guinness.* (19-year-old smoker)

7. Facilitators: Boys are more likely to smoke than girls and are often the entry point for their initial experimentation, such as by supplying shisha at a party.

Many girls view boys as more likely to take risks and “cooler” in a rebellious way, hence, more likely to try smoking. Girls are perceived as calmer and more likely to follow rules and, hence, less likely to start smoking voluntarily. Boys are also viewed as more likely to get around parental restrictions to access alcohol and other drugs, and therefore are perceived as more likely to use tobacco than girls.

*I think boys smoke more than girls*,* because boys are less likely to take advice than girls… and also*,* they hide it from their parents.* (16-year-old non-smoker)

The minority of girls who ever smoke usually do so socially and often in the company of boys who smoke. They are often first introduced to smoking by boys – both friends and romantic partners.

*My friend’s boyfriend brought the shisha and introduced it to me to have a taste of it*,* and I did. I did not think anything because shisha is not as hard as cigarettes.* (18-year-old experimenter)

*”Yes*,* I went to friend’s place and her boyfriend was smoking it [shisha] and I joined them… I just wanted to try and see. I was dull and dizzy like I have taken alcohol.* (17-year-old experimenter)

### Internal factors

8. Independence and desire to please: As girls grow older, they rely on more external sources of influence or make more independent decisions, but they still express a strong desire to behave in a way that pleases their parents.

Girls expand their sources of information as they grow older. They are more open to influence on lifestyle choices from visible external sources like celebrities and are more likely to claim independent decision-making, in comparison to younger girls who report high involvement of their parents. However, older girls are still guided by the basic values that their parents raised them with, and seek to behave in ways they would approve of. Girls of all ages consistently responded that out of everyone in their life they cared the most about pleasing their parents.

*Maame Serwaa*,* an actress [influences my choices when it comes to fashion*,* music*,* extra-curricular activities*,* and lifestyle in general] Her fashion sense*,* stature*,* I like… [I care the most about pleasing] my dad and mum. I want to give them the best.* (18-year-old non-smoker)

*I don’t have anyone [who influences my fashion and lifestyle choices]. I’m my own fashionista*,* I do my own thing. Whatever I think will look good I put it on.* (17-year-old non-smoker)

*No*,* I won’t do that [try smoking in the future]. It’s against my values. I wasn’t brought up like that.* (16-year-old non-smoker)

9. Romantic relationships are top of mind: Pressures to smoke and the negative risks involved are not top-of-mind concerns for non-smoking girls because they think they are unlikely to encounter it; rather, concerns around romantic relationships and the dating and sexual pressures that come with them are more salient to girls and their parents.

When asked what concerns are important to them, girls most often mention the pressures from older men to exchange sexual favors for material gains, or from boys to be romantically involved with them.

*[One of the biggest challenges that young girls face is] decision making*,* making decisions for yourself. One of my friends*,* if a guy proposes to say he loves her*,* she doesn’t have the boldness to say no.* (15-year-old non-smoker)

Girls also mention facing pressure from friends to accept favors from men, more than they mention feeling pressured to smoke, since most girls do not have smokers in their friend circles. Rather, they are more likely to have friends who are in intimate/romantic relationships with boys and men. Girls with low SES are especially vulnerable to these pressures, given the financial challenges that they face. Men, usually older, often offer material gifts like money, food, and airtime credit for phones in exchange for sex, which might seem attractive when a girl is struggling to afford those things.

*[If my friends asked me to do something that I was not comfortable with]*,* I would tell them no. Because I’m not interested in going out. I would worry that they would want a boy to have sex with me while I am drunk.* (15-year-old non-smoker)

Parents are also concerned about similar issues. Most parents expressed the fear of teenage pregnancy as their biggest concern for their daughters. This was highlighted since pregnancy could derail girls’ ability to stay in school and could ultimately affect their life outcomes.

*[My biggest worry for my child is] to fall into bad company*,* and not achieve what she wants. I just pray that she will listen to us [and avoid] prostitution*,* maybe early relationships*,* pregnancy and so on.* (Mother of 14-year old)

10. Untested confidence: Non-smoking girls express confidence in their ability to easily say no to smoking, yet most have never had the opportunity to actually do so. Although they admit it might be hard for others to say no to someone close to them, they believe personally they can stand their ground.

Girls generally expressed confidence in their ability to say no to things that they do not want, including smoking. Most, however, have never been offered tobacco before, and they believe that it is unlikely that people in their networks will ever ask them to smoke, given that most of their closest friends/family do not use tobacco.

*For me it’s easy [to say no to cigarettes] because I don’t like those things – the peer pressure… No [I have not said no before]*,* because I don’t have friends like that. Apart from school I don’t have friends. I don’t have any friends like that* (14-year-old non-smoker).

Many, however, admit that saying no to requests they are uncomfortable with from people in their closest networks will be difficult, especially when the person who is making the request is older than them.

*I’ll just make up an excuse*,* because I can’t say no straight to their face… Because they are your friends. You need them. So you can’t just say no straight in their face.* (13-year-old non-smoker)

11. Rationalizing shisha: Although few adolescent girls smoke, some can rationalize infrequent or social use of shisha because it is both seen as milder and more suitable for ladies than cigarettes, and they may perceive a social benefit.

Shisha is often the entry point for the minority of girls who experiment with tobacco. It is viewed by many girls as milder and less harmful than cigarettes and other forms of tobacco/drugs. They tend to like the flavored smell, including that it enables them to be more discreet about tobacco use, and therefore minimizes the social consequences that would come from smoking cigarettes. They may also find the act of smoking shisha attractive or appealing, and many girls see shisha as having fewer side effects than cigarettes.

*Cigarettes are more dangerous – they have more health effects. Shisha is designed in a flavor form so the effects are less but the substance used in cigarettes damages lungs faster.* (18-year-old non-smoker)

*It’s easy to detect when someone smokes cigarette unlike shisha*,* it is cool*,* secretive and you will never know that the person has smoked shisha. Yeah it has strawberry*,* vanilla and many different flavor and when you smoke it*,* it gives one a good breath.* (18-year-old non-smoker)

The communal dynamic of smoking shisha may contribute to the social benefits that some girls perceive, as they are often introduced to tobacco use by their friends or friends of friends. Girls from high-income households who smoke at parties and clubs are viewed as doing so for attention or to be seen as cool. Girls from low-income backgrounds who smoke may be relying on tobacco use as a coping mechanism through times of hardship. They often smoke with other girls in similar circumstances and enjoy the feeling of belonging to a social circle where they feel accepted.

*My friends introduced me into smoking of cigarettes*,* shisha*,* and it worked for me. The first time I felt dizzy but the second time everything was normal because I didn’t feel dizziness again*,* my friends always buy it for me. I feel happy whenever I take the shisha it makes you feel protected when you are with them for I like going out with friends and whenever there is [a risky or rowdy situation] they will protect you from any harm. It makes me feel happy each time they do that.* (18-year-old smoker)

Some girls describe smoking shisha as if it were an informed decision, and can rationalize the choice. They believe that smoking shisha infrequently limits the risks involved and by avoiding cigarettes and other forms of tobacco perceived to be stronger and more dangerous, they are avoiding most negative consequences. For them, the perceived social and personal benefits of using tobacco may outweigh the perceived minimal risks involved.

*Once a week [smoking shisha] is okay*,* using small is okay. It’s when the person takes it often*,* like 5 times a week that it’s a problem.* (14-year-old non-smoker)

## Discussion

We conducted this qualitative research to explore the exposure to and perceptions of tobacco use among teenage girls in Ghana and to understand the social, physical, and psychological context underlying their smoking and tobacco use behavior. This understanding will be important for efforts to develop age- and gender-specific local tobacco prevention programming. While our findings in many ways align with the global literature on adolescent tobacco use, including associations between age and social exposure [36], here we present a more nuanced picture of the specific influencing factors behind those trends in Ghana – related to social influences on perceptions, environmental opportunity factors, and personal decision-making factors. Compared to other countries with a higher burden of tobacco, smoking rates among both adults and adolescents in Ghana are considered quite low [45], although the recent increases in youth use and narrowing gender gap observed among students [15] suggest that adolescent girls are a particularly important population to study. The few Ghana studies published have used survey data to examine general trends in youth use and demographic associations [7, 17, 34, 46], but have not qualitatively explored the behavioral context for both smoking and non-smoking among adolescent girls or considered what might make currently non-smoking girls vulnerable to future smoking. Our research sought to fill this gap.

Our findings underscore that perceptions around smoking are very much driven by girls’ social world, which is generally small and parent-centric for non-smokers at younger ages but can start to expand as girls get farther along in school or leave school entirely. The literature suggests the importance of parental influence, particularly the significant risk factor of parental smoking for youth smoking [7], but our results highlight the pivotal protective role the parental relationship plays at younger ages and even at older ages as girls express desires to continue living up to their parents’ values. In many ways, girls in Ghana are relatively sheltered, as watchful parents restrict their movement in public and their interactions with others, and they generally have a small number of friends from similar backgrounds. At a young age, without any personal connection to smokers or smoking, negative perceptions are driven by prominent social beliefs about moral failure shaped by the values of parents and close friends and reinforced by general awareness of health-related risks.

This influence of girls’ closest social circles over other peers is consistent with the literature; for example, a meta-analysis study found that the sexual behavior of close friends was more influential on adolescent sexual behavior than that of more distant acquaintances like unspecified school peers [47]. Much has been written about the particular vulnerability of adolescents to peer pressure [29], but our findings emphasize the positive role of social influence as an inhibitor of smoking among young girls. It is only the small segment of the population that does not feel this pressure, often due to lack of family oversight, that is willing to partake in perceived negative behaviors. This is also consistent with the literature on social norms, which in this context reflect strong moral judgments on behavior related to smoking, often with religious undertones given the prominence of religion in Ghanaian culture [48].

As girls progress through schooling and emerge from a relatively sheltered childhood, their social world expands. This may involve new opportunities or pressures that they had not previously envisioned being confronted with and in some ways are unprepared for managing after having been so aligned on values with their tight social circle earlier in life. Consistent with the global literature, rates of adolescent smoking in Ghana increase with age, and our findings highlight how this reflects a changing social context. Studies have found that as children grow older, parents monitor and control them less, which can sometimes expose the children to more risky situations [49–51]. The primary concerns cited by girls and their parents during interviews were related to intimate relationships and the fear of being taken advantage of by older men. The relative prominence of this issue means that less salient possibilities, such as being confronted with the opportunity to smoke, do not receive the same attention or consideration as a problem to anticipate. Interview participants at first expressed high confidence that they would turn down an offer of smoking, since it seems like the obvious choice given their current attitudes towards the behavior; however, it is not a position that they ever considered they might be in. Only once probed about how they would feel turning down someone close to them do they start to imagine the potential difficulty. This reflects a form of projection bias [52] in which people overestimate how much their future selves will hold the same beliefs and behaviors as their current selves and aligns with studies that show that adolescents, like adults, are often overconfident in unfamiliar situations, and under-confident in situations with which they are more familiar [53]. Adolescents get better at decision-making when they have had prior opportunities to use such skills [54, 55], but they remain at risk of “danger invulnerability,” in which they are more vulnerable to negative consequences when they don’t think they are likely to face a certain situation [56].

Studies in the United States have linked smoking among adolescent girls to an expression of their independence [32]. Here we find that many older girls in Ghana also look for ways to express their independence in decision-making after what may have been a sheltered adolescence, whereas the few girls with less protective families may have started making their own choices at earlier ages. Adolescent vulnerability to peer pressure is cited as a risk factor for smoking [20], but even without overt pressure, under the right conditions, girls may perceive reduced consequences and even a social benefit to partaking in an activity like smoking shisha. In that case, they may use their developing agency to rationalize limited or infrequent use as a way of maintaining control over the cost-benefit calculation so that it still works out in their favor. Shisha is often seen by girls as different from cigarettes; the danger of shisha use is often a matter of degree. While limited or infrequent use is not as damaging as chronic smoking behavior, experimentation during youth is associated with a higher risk of long-term use and could be the entry point to other tobacco products [57]. This overemphasis on the benefits and de-emphasis on risks is in line with past studies that show that adolescents are more prone to focusing on incentives and rewards over risks [58] and more likely to pay attention to immediate consequences of their actions rather than the long term effects [59]. As adolescents are particularly sensitive to social incentives, especially with regard to the rewards of socializing with peers, they may be driven to act in ways that they believe will gain them the admiration of their friends [60], sometimes, even against their or others’ wishes or values. This may be especially true in situations that are emotionally or socially arousing, where adolescents may find it more difficult to exercise self-control [61]. Understanding the mental calculations that girls are making and the influences on their decisions and actions will be important to any prevention efforts.

There are several limitations of our study. Interview participants were recruited purposively to represent a mix of perspectives and so were not intended to be representative of the adolescent girl population in the study area. For example, we wanted to include the perspectives of adolescent smokers, and so oversampled them for interviews and to help validate our survey instrument for a future intervention study.Yet the overall sample still reflects that the vast majority of girls are non-smoking; including their various perspectives and experiences helps contribute to a more comprehensive understanding of how context influences risk and behavior. Another challenge is that when asking about sensitive topics, such as smoking behavior in a context where it is perceived to be highly disapproved of, participants may give responses that they think will make them look better. While we cannot entirely rule out this possibility of social desirability bias, we made sure to protect the privacy of participants and validated the reliability of self-reported smoking behavior in this population using saliva nicotine tests during a secondary survey, at least within the most recent 48-hour recall window that the biomarker test would capture. Another potential limitation is that the study activities were conducted during the first year of the COVID-19 pandemic, and so safety precautions including the use of masks and social distancing may have hindered the ability of interviewers and enumerators to build rapport with the young participants, thus impacting the quality of responses. However, the study’s reliance on primarily young female researchers to conduct interviews might have countered this by helping the participants feel more comfortable with the discussion. Finally, conducting three rounds of data collection, between which the interview guides were adjusted, could risk reducing the comparability of results across rounds. However, an advantage of this approach is that it allows for adaptability in the research process; during the breaks researchers were able to reflect on initial insights and identify which perspectives and lines of inquiry needed additional investigation, leading to a more comprehensive and nuanced understanding of the research topic as well as a wider range of perspectives.

## Conclusion

Rising tobacco use among adolescents in LMICs and narrowing gender gaps for tobacco uptake in many countries suggest that adolescent girls are a priority population for smoking prevention efforts and that gender-specific interventions to reduce smoking initiation may be warranted. We sought to develop a more nuanced understanding of the behavioral context for both smoking and non-smoking among adolescent girls in urban Ghana to identify influencing factors and consider risks for future smoking. Our findings highlight strong social influences on perceptions, exposure to opportunities, and decision-making in ways that can often be protective against smoking, particularly at younger ages, but can also leave girls unprepared to manage challenging situations later in life. Leveraging these protective factors while considering ways to reshape girls’ social environments in ways that limit opportunities to smoke could be promising opportunities for prevention. Understanding this context in Ghana will be important for any future programming aiming to address smoking behavior among adolescents.

## Electronic supplementary material

Below is the link to the electronic supplementary material.

Supplementary Material 1

## Data Availability

The data that support the findings of this study are not publicly available due to privacy and ethical restrictions but are available from the corresponding author on reasonable request.

## Abbreviations

IRB: Institutional Review Board
JHS: Junior High School
LMIC: Low and middle-income countries
SES: Socioeconomic status
SHS: Senior High School
