# Supplementary material for: Social norms and social opportunities: a qualitative study of influences on tobacco use among urban adolescent girls in Ghana
Source: BMC Public Health. 2024 Oct 28;24:2978. doi: 10.1186/s12889-024-20413-z (PMC11514744; doi:10.1186/s12889-024-20413-z)
Supplement: Supplementary file 1 — Supplementary Material 1 [file 12889_2024_20413_MOESM1_ESM.pdf]

INTERVIEW GUIDE

I: Introduction

1. Could you tell me a little bit about yourself?
   a. How old are you?
   b. Who do you live with?
   c. Are you in school?
   d. [If yes] What school do you go to?
      i. Public/private
      ii. JHS/SHS
   e. [If yes] What class are you in?

II: Peer groups

2. Please tell me a bit about your friends. Who are your 5 closest friends?
   a. For how long have you been friends?
   b. How did you become friends with this group of girls?
   c. Do you go to the same school as these friends? If no, do you have friends at school?
   d. Do you live in the same neighborhood as these friends? If no, do you have friends in your neighborhood?
   e. What are some things that you have in common with your friends (ask for friends at school, and then in your neighborhood)?
   f. What is the thing you most admire about your 5 closest friends?
   g. What is the most important quality you look for when choosing a friend?
3. What do you and your friends typically do together?
   a. When do you spend time with your friends? (probe: in vs. out of school, weekdays vs. weekends)
   b. Where do you spend time with your friends?
   c. What activities do you do with your friends?
   d. What do you talk with your friends about? On what topics, if any, do your friends advise you?
   e. [For SHS girls only] What do you do with your friends now that you didn't do in JHS?
   f. [For SHS girls only] What activities are girls exposed to in SHS that they weren't exposed to in JHS?
4. [For SHS girls only] Do you have the same group of friends as you had in JHS?
   a. [If no] what are some reasons you made new friends?
   b. How did you become friends with your friends in JHS? What about SHS?
   c. How have your activities changed since JHS?
5. Have any of your friends ever engaged in an activity or lifestyle that you do not feel comfortable with?
   a. If yes, what was it that made you feel uncomfortable?
   b. If yes, how did you respond?
   c. What would you do if your friends asked you to do something you weren't comfortable with? Why would you react this way? How would your friends respond?
   d. What, if anything, could make it easier to say "no" to a friend?
   e. Have you ever felt pressured by your friends to do something you weren't comfortable with? Tell me about the experience.

6. *Outside* of your current group of friends, who in your school or neighborhood would you most like to be friends with? Why would you like to be friends with this person?
7. How would you describe someone who is cool?
    a. Who is the coolest (most admired) person in your school (/community if not in school)?
        i. What makes them cool?
        ii. What kind of activities are cool people likely to engage in (inside and outside of school)?
        iii. What kind of activities are cool people NOT likely to engage in (inside and outside of school)?
        iv. Is a cool person likely or unlikely to smoke, use shisha or other tobacco products?
            1. Why?
            2. Which product are they likely to use?
    b. Which person would you like to be like when you grow up?
        i. Why would you like to be like them?
        ii. Does this person/Is this person likely to smoke, use shisha or other tobacco products?
        iii. How do you think they would view smoking or using shisha?
8. Who in your life do you care the most about pleasing?
    a. Who would you say influences your choices when it comes to fashion, music, extra-curricular activities, and lifestyle in general? Why?
    b. Who would you be happy to get approval from when it comes to music, fashion, extra-curricular activities, and lifestyle in general? Why?
    c. Whose advice do you listen to the most? Why?
        i. What kind of topics does this person advise you on?
    d. Do any of these people (or are any of them likely to) smoke, use shisha, or use other tobacco products?
        i. Which of them? Why do you think so?
    e. Are any of these people unlikely to smoke, use shisha, or use other tobacco products?
        i. Which of them? Why do you think so?

III: Parental influence

9. [For SHS girls] Who are you most likely to go to for advice?
    a. Why do you turn to this person for advice?
    b. What does this person advise you on? Is there anything that you could not ask this person about? What are some of the things you cannot ask this person about?
10. How much time do you spend with your parents? **[If she doesn't live with her parents, skip to Q16]**
    a. How has this changed as you've gotten older?
    b. What are some things your parents do with you? What are some things your parents do without you?
    c. What are some things you do when your parents are around? What are some things you do when you parents aren't around?
11. How likely are you to go to your parents when you want advice?
    a. What kinds of things can you talk to your parents about?

    b. What kinds of things can you NOT talk to your parents about? Who are you likely to talk to instead?
12. [For SHS girls] How (if at all) has your relationship changed with your parents as you've gotten older?
    a. Are you more or less likely to go to your parents for advice compared to when you were in JHS?
    b. What are some things you are more likely to talk to discuss with your parents? Less likely?
13. How much do your parents influence which activities you do?
    a. How much do your parents influence your choice of friends?
    b. [For SHS girls] Do your parents influence your choices more or less compared to when you were in JHS?
14. How often do your parents allow you to spend time with your friends?
    a. What activities do you do with your friends that your parents approve of?
    b. What is one thing you do that your parents do not approve of?
    c. How do you respond if your parents tell you not to do something that your friends want you to do? Why?
    d. Who do your parents allow you to spend time with? Why?
    e. Who do your parents not allow you to spend time with? Why?
15. Do your parents ever give you money?
    a. [If yes] What do they allow you/advise you to spend it on?
    b. What do you typically spend this money on?
16. [If parents are not around] For how long have you been away from your parents?
    a. What was the situation that caused you to stop spending time with your parents?
    b. Who do you live with/spend time with instead?
    c. How has your life changed since you stopped living with/spending time with your parents?

IV: Influence from Boys

17. How common is it for girls your age to have boyfriends or romantic partners?
    a. Do girls your age typically date boys who are the same age or older/younger?
    b. How common is it for girls your age to have a partner who is more than 5 years older than her?
18. Do any of your friends have boyfriends or romantic partners?
    a. [If yes] how did they meet their partner?
    b. [If yes] what kinds of activities do they do with their boyfriends?
    c. What kinds of activities do boys like to do with their girlfriends?
    d. Have you ever heard of one of your friends' boyfriends pressuring her to do something she didn't want to do? What did he ask her to do? How did she respond?
19. Do you have a boyfriend or romantic partner?
    a. [If yes] What does your boyfriend/partner do on weekends? Where does he spend time? How often do you spend time with him there?
    b. [If yes] What kinds of activities do you and your boyfriend do together?
    c. How (if at all) has your life changed since you started dating your boyfriend/hanging out with your partner?
    d. [If you feel comfortable answering] Has your boyfriend/partner ever asked you to do something or go somewhere that you didn't feel comfortable with? What did he ask you to do?
    e. Does your boyfriend/partner engage in any activities or lifestyles that you do not agree with? If so, what are these?

V: Smoking

20. Do you and/or your friends ever go to parties or bars?
    a. [If yes] What do you do there?
    b. [If yes] When do you go?
    c. [If yes] At what age did you start going to parties/bars?
    d. [If yes] Who did you first go to parties/bars with? What was the occasion (if any)?
    e. [If yes] What kinds of people usually go to parties/bars?
    f. Do your parents know that you go to parties/bars? What do they think?
21. Have any of your friends tried drinking alcohol?
    a. [If yes] Do they do it openly? Who do they hide it from, if anyone?
    b. [If yes] Do they ever drink when you are around?
    c. [If yes] Did they ask you to join/try drinking?
    d. [If yes] Did you join them? Why or why not?
    e. [If no] What would you do if your best friend asked you to drink with her? Why would you react that way?
22. What is shisha?
23. Have any of your friends tried smoking a cigarette? Shisha?
    a. [If yes] Do they do it openly? Who do they hide it from, if anyone?
    b. [If yes] Do they ever smoke when you are around?
    c. [If yes] Did they ask you to join/try smoking?
    d. [If yes] Did you join them? Why or why not?
    e. [If no] What would you do if your best friend asked you to smoke with her? Why would you react that way?
24. [If girl has boyfriend] Has your boyfriend ever tried smoking a cigarette? Shisha?
    a. [If yes] does he ever smoke when you are around?
    b. [If yes] Did he ask you to join/try smoking?
    c. [If yes] Did you join him? Why or why not?
    d. [If no] What would you do if your boyfriend asked you to smoke with him? Why would you react that way?
25. What kinds of girls are most likely to smoke cigarettes? Shisha? [probe: age, education, school status, SES, personality, popularity]
    a. What do girls think of other girls who smoke cigarettes? Shisha?
    b. What do boys think of girls who smoke cigarettes? Shisha?
    c. What do *your friends* think of girls who smoke cigarettes? Shisha?
26. Are there differences between boys and girls when it comes to smoking/ shisha/tobacco use?
    a. Are boys or girls more likely to smoke, use shisha or other tobacco products?
    b. Are boys more likely to use certain products than girls (and vice-versa)?
        i. Which products are boys more likely to use?
        ii. Which products are girls more likely to use?
    c. What do girls your age think about boys who smoke/use shisha/tobacco products?
    d. What do boys your age think about girls who smoke/use shisha/other tobacco products?
27. How much smoking/shisha/tobacco use is too much?
    a. Is there a level/frequency beyond which it becomes unsafe to use?
    b. Are there differences in the risk of smoking/using shisha/other tobacco products?
        i. Which products do you consider more safe?
        ii. Which products do you consider more dangerous?

28. Do you know people who smoke/use shisha/other tobacco products?
    a. What kinds of people use shisha/tobacco/smoke? (Probe: age, gender, personality)
    b. Why do these people use shisha/tobacco/smoke?
    c. Which products do they smoke/use?
    d. How often do they smoke/use these products?
    e. Who do they typically smoke with?
    f. What do other people think about people who smoke/use tobacco?
29. How common do you think smoking/using shisha/other tobacco products is (probe very common, very rare, or somewhere in the middle)?
    a. When do people smoke/use shisha/other products the most?
    b. What are some reasons that people might use tobacco?
    c. What benefits do they derive from it?
    d. What disadvantages do they face from tobacco use?
30. Has smoking/shisha/tobacco use changed due to COVID-19?
    a. If yes, how so? Probe: Frequency, location, who it's done with, how it's done/changed practices, etc.
31. I would like to learn about whether or not there are any social consequences to smoking/shisha/tobacco use or abstinence from it.
    a. When other girls your age smoke/use shisha/ tobacco, how do others view/treat them?
    b. When girls your age DO NOT smoke/use shisha/ tobacco, how do others view/treat them?
    c. If you smoked/used shisha/tobacco products, how would your friends view/treat you?
    d. If you did not smoke/use shisha/tobacco products, how would your friends view/treat you?
    e. Do you know of any cool people (who are admired by their peers) who do not smoke/use shisha/other tobacco products or have said no when offered these products?
        i. How do you view such people?
        ii. How do others view them?
32. How do people get access to shisha/tobacco products?
33. Do you have the freedom to decide whether or not to smoke/use shisha/tobacco products?
    a. If you do not want to use smoke/use shisha/tobacco products, what is the best way to avoid them?
    b. If you do not want to smoke/use shisha/ tobacco products, what is the best thing to do/say when your friends offer them to you?
    c. Is it cool to say no when you're offered shisha/tobacco products?
    d. When is it difficult to say no/what makes it difficult to say no when you are invited to smoke/use shisha/tobacco?
    e. What makes it easy to say no?
    f. Have you said no to smoking/shisha/tobacco use before?
        i. How did you feel afterward?
34. Have you ever smoked a cigarette? Shisha?
    a. [If yes] How did your friends react when you started smoking?
    b. [If no] What would your friends think about you if you started smoking?
    c. [If no] How likely do you think you are to try smoking in the future? Why is that?
35. [If tried] When did you smoke/use shisha/tobacco products for the first time?
    a. How were you introduced to smoking/shisha/tobacco products?
        i. [If by a person] Who introduced you to smoking?

ii. What is your relationship with them?
iii. What do you remember about that time?
iv. How did you feel?

b. What are some reasons you decided to try smoking/using shisha/tobacco products the first time that you did?
c. On which days of the week do you typically smoke/use shisha/other tobacco products?
d. On which occasions do you typically smoke/use shisha/other tobacco places?
e. Where do you typically smoke/use shisha/other tobacco products?
f. Who are you usually with when you smoke/use shisha/other tobacco products?
g. Have you thought about stopping smoking/using shisha/tobacco?
    i. If yes, what are some reasons that would cause you to stop?

- Thank you once again for meeting with us, and sharing your experiences.
- Is there anything I should have asked that I didn't?
- Anything else you would like to share? Or any other questions that you have for us?
- Once again, thank you for sharing your perspectives with us.
